# Clinical relevance of B7H3 expression in retinoblastoma

**DOI:** 10.1038/s41598-020-67101-7

**Published:** 2020-06-23

**Authors:** Bhuvaneswari Ganesan, Sowmya Parameswaran, Ashwani Sharma, Subramanian Krishnakumar

**Affiliations:** 1 L&T Ocular Pathology Department, Vision Research Foundation, Chennai, India; 2 Radheshyam Kanoi Stem Cell Laboratory, Vision Research Foundation, Chennai, India; 3 Department of Chemistry & Biology, Indian Institute of Science Education and Research (IISER), Tirupati, India

**Keywords:** Cancer, Immunology, Medical research

## Abstract

Retinoblastoma (RB) is the most common paediatric intraocular tumour. Currently, chemotherapy is widely used to reduce the chance of metastasis as well as for vision salvage. The limitations of chemotherapy for RB include chemoresistance and cytotoxicity. Recently, immunotherapy is considered for treating chemoresistant cancers. Although, several molecular targets are available for immunotherapy in different cancers, we were interested in B7H3, as it was differentially expressed between retinoblastoma and retina in our earlier proteomics study. Hence, in this study we validated the previous finding by Western blotting and immunohistochemistry on primary RB tumor samples. The results suggest significantly increased expression of B7H3 in RB tumor samples compared to retina by western blotting. Immunohistochemistry revealed spatial, inter and intratumoral heterogeneity in the primary RB tumor sections. Correlation of the B7H3 expression with clinical and histopathological data revealed significantly increased expression of B7H3 in poorly differentiated, non-neural invasive tumors and lower expression in neural invasion and severe anaplastic areas of the tumors. B7H3 expression did not significantly vary between low-risk and high-risk tumors. The study also revealed considerably reduced infiltration of T lymphocytes in RB. We conclude that B7H3 is prominently expressed in primary RB tumors and could be used for targeted therapy.

## Introduction

Retinoblastoma (RB) is the most common primary intraocular tumor in childhood. Apart from the retinoblastoma gene (RB1), v-myc myelocytomatosis viral related oncogene, neuroblastoma derived homolog (MYCN), and other somatic genetic aberrations have been attributed to tumor progression. Currently, focal and systemic chemotherapy is being used for treating RB. Although it is safe and effective in majority of the cases, chemotoxicity and chemoresistance are being reported^1–3^. Recently, immunotherapy is gaining momentum in treating tumors including chemoresistant RB. Several monoclonal antibody based cancer immunotherapy molecules have been approved recently by Food and Drug Administration (FDA). This includes Programmed death-ligand 1 (PDL1), Programmed cell death protein 1 (PD1), cytotoxic T-lymphocyte-associated protein 4 (CTLA4), CD20 and CD54 for different cancers. Of these, both PDL1 and PD1 belong to the B7 family checkpoint molecule and their expression has been recently evaluated in RB tumour^4,5^. Other than the B7 family, an *in vitro* study using Spleen Tyrosine Kinase (SYK) targeted dendritic cell based immunotherapy was shown to be effective in chemoresistant RB^6^.

We had earlier performed a membrane proteomics comparing primary retinoblastoma and retinal tissue^7^. While comparing their expression for immunotherapy molecules we found B7-H3 (CD276); one of the B7 family checkpoint molecule to be overexpressed in RB tumor compared to the retinal tissue. Since, B7H3 is known to be expressed in several malignant tumors with over expression correlating with significant risk of metastasis and/or poor prognosis^8–17^ we wanted to evaluate it in primary RB tumors. In addition, since B7H3 is known to play a role as co-activator or co-inhibitor of T lymphocytes, we also evaluated the distribution T lymphocytes using the markers CD3; CD4 and CD8 in the context of B7H3 expression in primary RB tumors.

## Results

### B7H3 expression in retinoblastoma and in retina by immunoblotting

**Figure 1:**
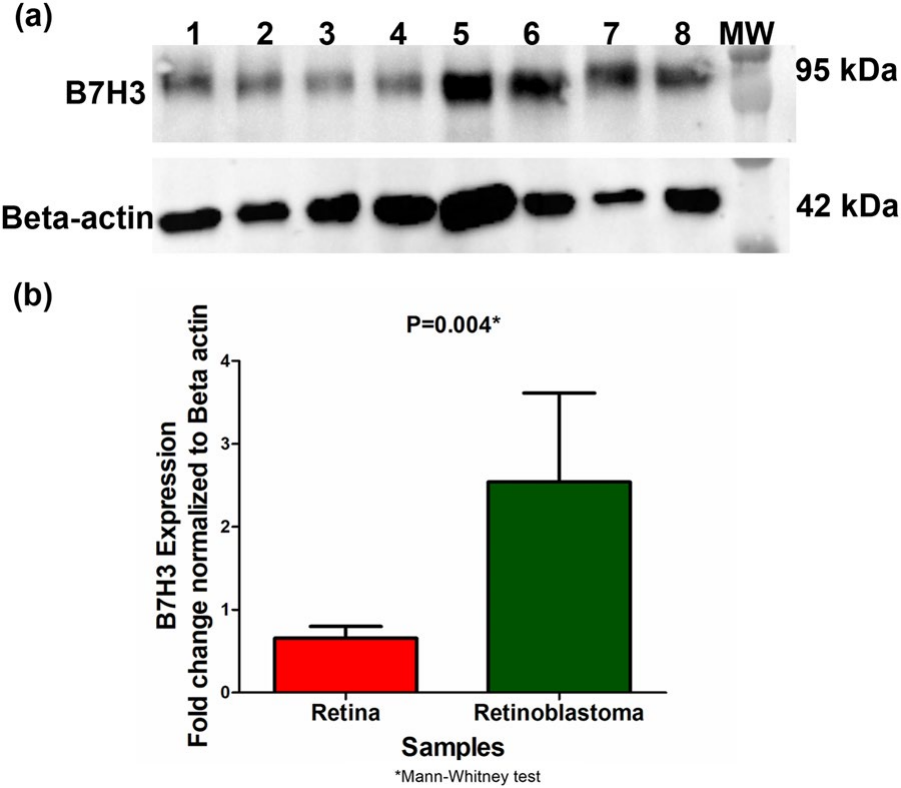
Expression of B7H3 in primary RB tumour and retina samples. Immunoblot shows B7H3 protein expression in primary RB tumour(n = 8)(lane 5–8); Retina normal cadaveric tissue(n = 4) (lane 1–4) (**a**). B7H3 expression was significantly higher in primary RB tumour sample compared to retina (p = 0.004)(Mann Whitney Test) (**b**).

Western blotting was performed on protein lysates of RB tumors (n = 8) and normal cadaveric retina (n = 4) for B7H3 (90–110 kDa). B7H3 expression was significantly increased in RB tumour compared to normal retina (p = 0.004) (Mann-Whitney test) (Fig. 1a,b; Supplementary material Fig. S1).

### B7H3 Expression in human cadaveric retina by immunohistochemistry

B7H3 expression was examined by immunohistochemistry(IHC) in two retinal tissue obtained from cadaveric eyes. The intensity of B7H3 was appreciated highly in the outer nuclear layer of the retina (Supplementary Fig. S2).

### B7H3 is expressed in retinoblastoma tumor by immunohistochemistry

**Figure 2:**
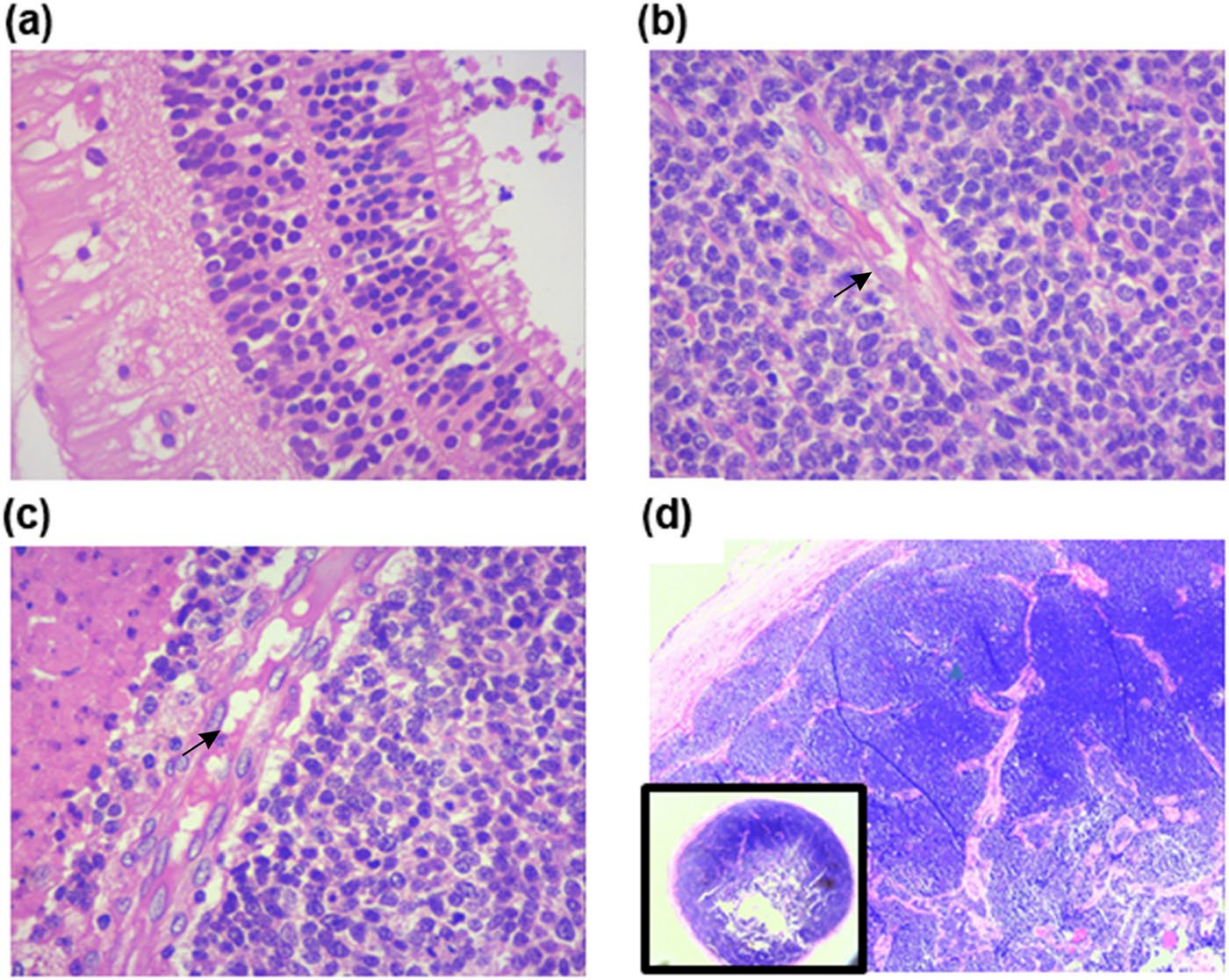
H & E Images of : Retina, tumour lobule, blood vessel, surgical end of optic nerve. (**a**) Photomicrograph shows portion of retinal tissue adjacent to RB tumour. (**b**) Tumour lobules of RB arranged around the blood vessels. (**c**) Blood vessels surrounded by tumour cells. Black arrows indicate blood vessels. (**d**) Surgical end of the optic nerve invaded by RB tumour cells insert showing low magnification of the surgical end of the optic nerve.

**Figure 3:**
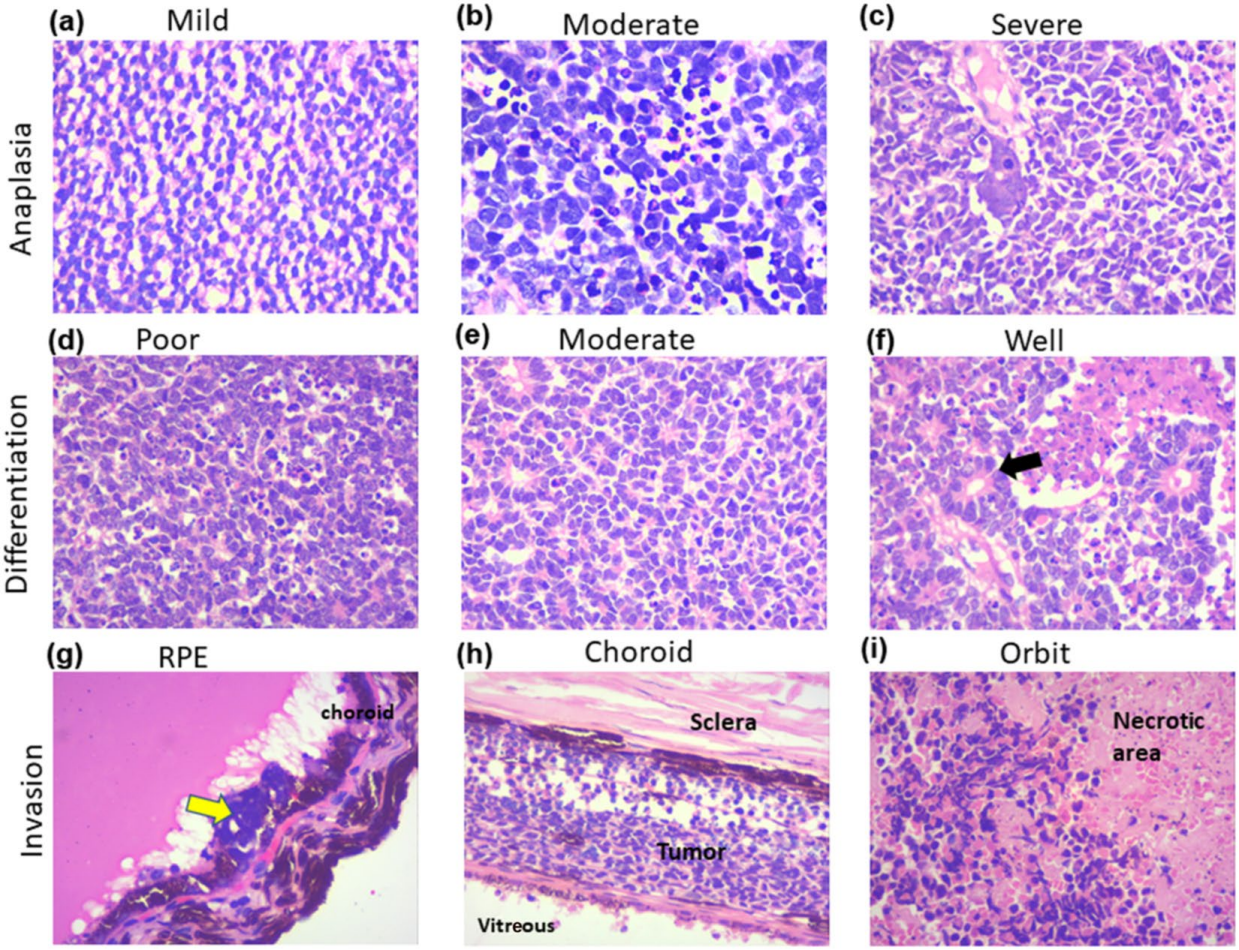
H& E Images of primary retinoblastoma tumors. H and E staining of primary retinoblastoma tumors showed different grades of anaplasia viz: mild, moderate, severe (**a**–**c**). Primary RB tumors based on their differentiation status were classified either as poor, moderate or well differentiated (**d**–**f**). The RB tumors invaded non- neural structures such as RPE, choroid and orbit (**g**–**i**). Black arrow (in panel f) shows Flexner wintersteiner rosettes in a well differentiated primary RB tumour. Yellow arrow (in panel g) shows tumour cells invading RPE.

**Figure 4:**
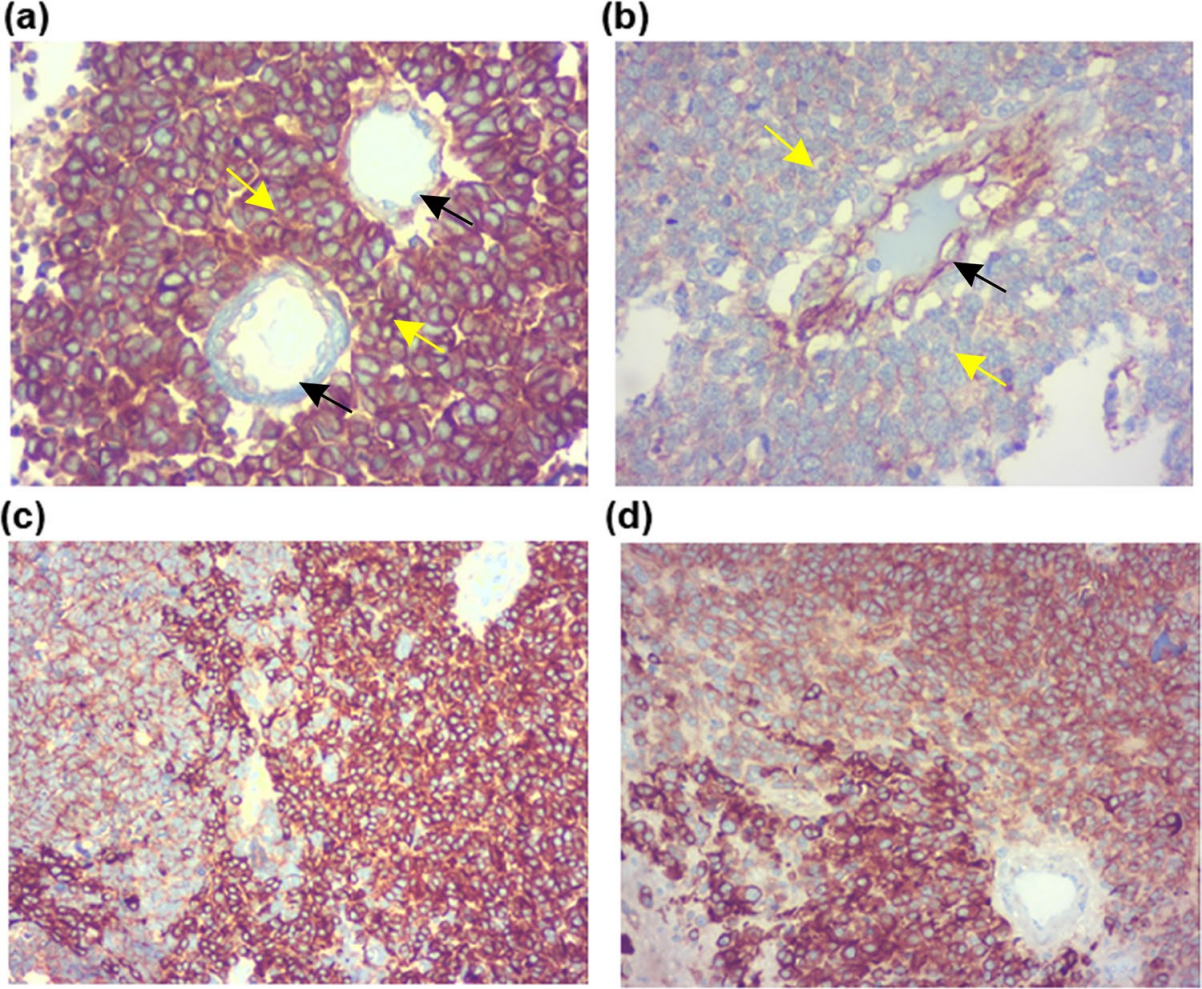
Heterogenous expression of B7H3 in Tumour lobules and Blood vessels. Strong expression in (IRS score=12) tumour lobules with weak expression in blood vessel (**a**). Strong expression in blood vessel with weak expression of B7H3 in tumour lobule (IRS score = 4(b). Heterogenous expression of B7H3 within the tumour lobule. (IRS score = 9(c), 6(d)). Black arrows indicate blood vessels, Yellow arrows indicate tumor lobules.

In order to assess the expression of B7H3 in the primary RB tumor, the regions of tumor were spatially and histologically classified by hematoxylin and eosin (H and E) staining. Enucleated eye of the primary RB tumour contained retina, tumor lobules (where tumor cells are arranged around the blood vessels), tumour blood vessels, surgical end of optic nerve (Fig. 2), regions of anaplasia-mild, moderate and severe (Fig. 3a–c); differentiation- poor, moderate, well (Fig. 3d–f); invasion- retinal pigment epithelium (RPE), choroid, and orbit (Fig. 3g–i). Immunohistochemistry for B7H3 was carried out on breast cancer (positive control) and Non-Hodgkin’s lymphoma (Negative control) for its validation (Supplementary material Fig. S3a). In case of RB tumor samples, B7H3 was expressed in all 35 samples. However, the intensity of the expression varied between tumor lobules and blood vessels (Fig. 4a,b) as well as within the individual tumour (Fig. 4c,d).

A total of 64 blood vessel regions of 20 RB tumors were analysed for B7H3 expression patterns. The analysis revealed that whenever the B7H3 expression was stronger in the tumour lobules the expression was weaker in the blood vessels and vice versa (Fig. 4a,b).

**Figure 5:**
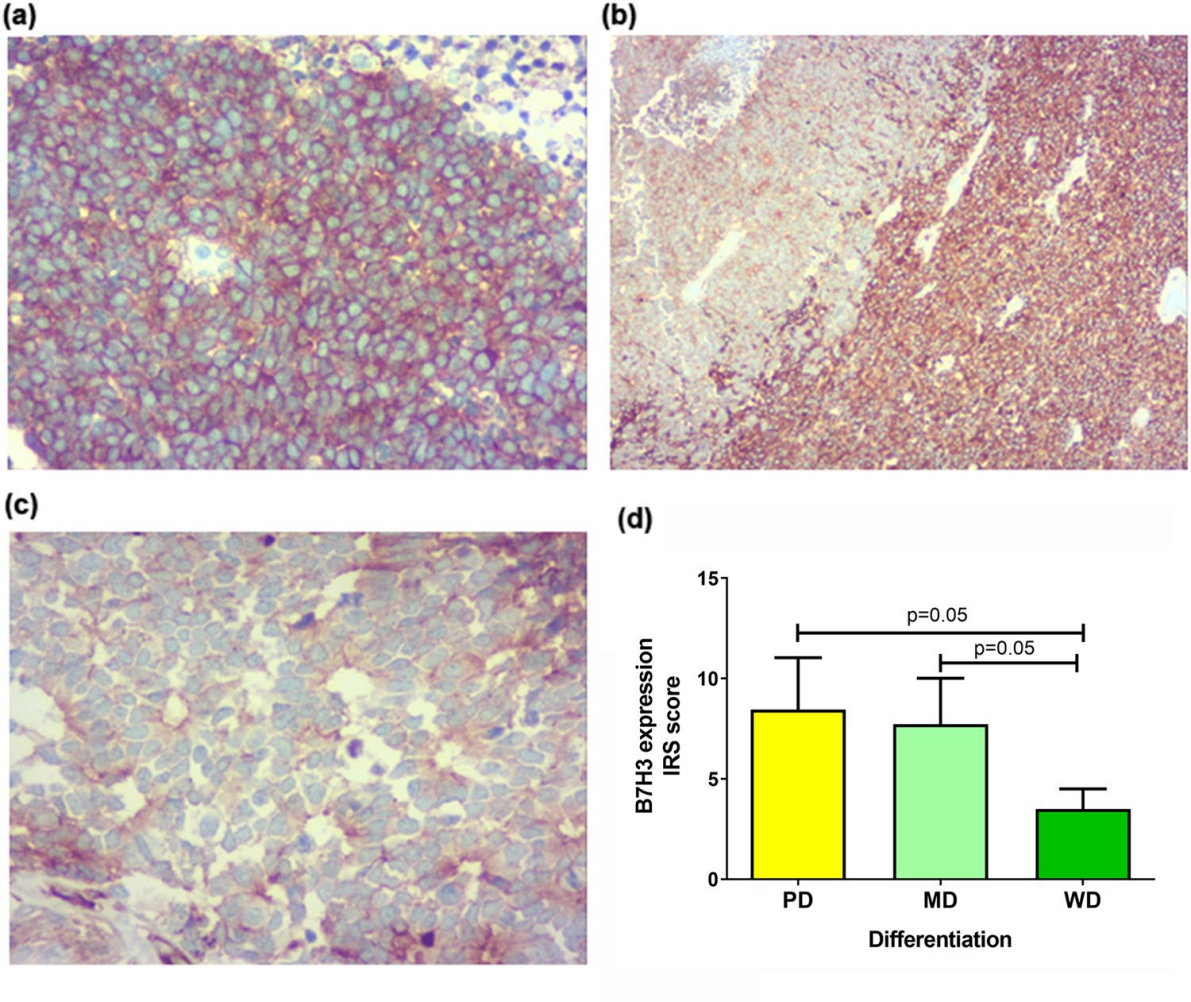
Expression of B7H3 in relation to differentiation status of retinoblastoma. B7H3 expression was significantly increased PD RB tumour (**a**) (IRS score mean ± SD)(8.45 ± 2.58) compared to moderately(7.27 ± 2.28) (**b**) and well differentiated(3.50 ± 1.00) (**c**) RB tumour, (p = 0.005) (**d**) (ANOVA).

B7H3 expression in RB was analysed with different pathological features. B7H3 expression (mean IRS score ±SD) was significantly increased in poorly differentiated (8.45 ± 2.58; n = 20) RB tumours (p = 0.005.) (Kruskal-Wallis test) than the moderately differentiated (7.72 ± 2.28; n = 11) and well differentiated tumours (3.5 ± 1; n = 4), (Fig. 5).

**Figure 6:**
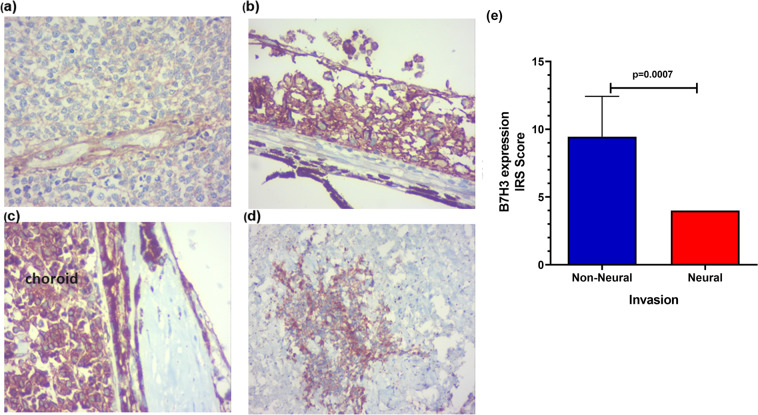
B7H3 expression in relation to invasion status of RB. B7H3 expression was significantly decreased in neural invasion (optic nerve) (**a**) compared to the non-neural tissue (RPE (**b**), Choroid(**c**), Orbit(**d**) (p = 0.0007) (**e**).

While comparing the B7H3 expression (mean IRS score ± SD) in the regions of neural (optic nerve) and non-neural (RPE, choroidal, scleral/Orbital), invasion sites, significant decrease was noted in neural invasion (4.0 ± 0.0; n = 4)compared to non-neural (9.45 ± 2.97;n = 11) (p = 0.0007)(Mann Whitney Test) (Fig. 6).

**Figure 7:**
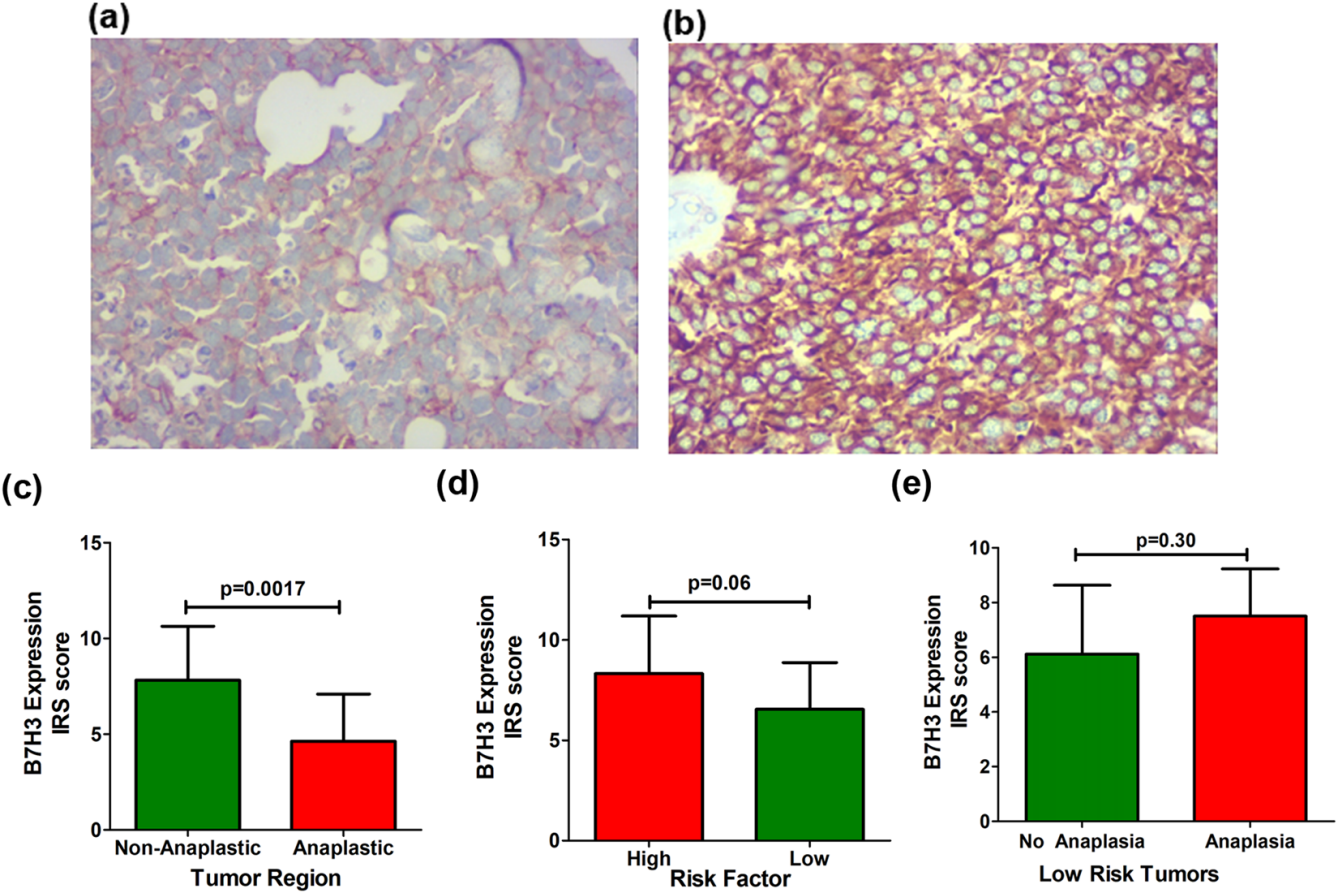
B7H3 Expression in relation to Anaplasia. Comparative expression of B7H3 in anaplastic (**a**) and non-anaplastic area (**b**) of the same tumour. Anaplastic area had weaker B7H3 expression than the non-anaplastic area, p = 0.0017(**c)**. B7H3 expression in relation to high and low risk tumors: B7H3 expression did not significantly differ between the high and low risk tumor. p = 0.06 (**d**). B7H3 expression in relation to low risk tumors with and without severe anaplasia: B7H3 expression did not differ between the low risk tumors with and without severe anaplasia, p = 0.30(**e**).

B7H3 expression (mean IRS score ± SD) in anaplastic regions (4.63 ± 2.46; n = 11) (Fig. 7a) showed significant decrease compared to non-anaplastic regions (7.8 ± 2.82; n = 11) (Fig. 7b) of the same tumor (p = 0.0017; paired t test) (Fig. 7c). B7H3 expression (mean IRS score ±SD) did not show any significant difference between high risk (8.31 ± 2.86; n = 22) and low risk (6.53 ± 2.33; n = 13) tumors (p = 0.06; unpaired t Test) (Fig. 7d). In addition, B7H3 expression also did not significantly differ between the low-risk tumors with and without severe anaplasia (p = 0.36; Mann Whitney Test) (Fig. 7e).

### T cell markers CD3, CD4, CD8, FOXP3 in Retinoblastoma

Since, B7H3 is reported to decrease T lymphocyte recruitment into the tumour regions, we analysed the T lymphocytes distribution in different regions of RB tumour. H and E staining on primary RB tumour did not reveal a clear inflammatory component except for a few macrophages^18^. Hence, specific T lymphocyte markers such as CD3,CD4,CD8 were carried out on RB tumors (n = 12) and different regions (Tumor lobule, tumor blood vessels, Retinal blood vessel, invading front, Necrotic area) were analysed. In order to validate, the T lymphocyte marker expression, tonsil tissue was used as a positive control (Supplementary material Fig. S3b).

**Figure 8:**
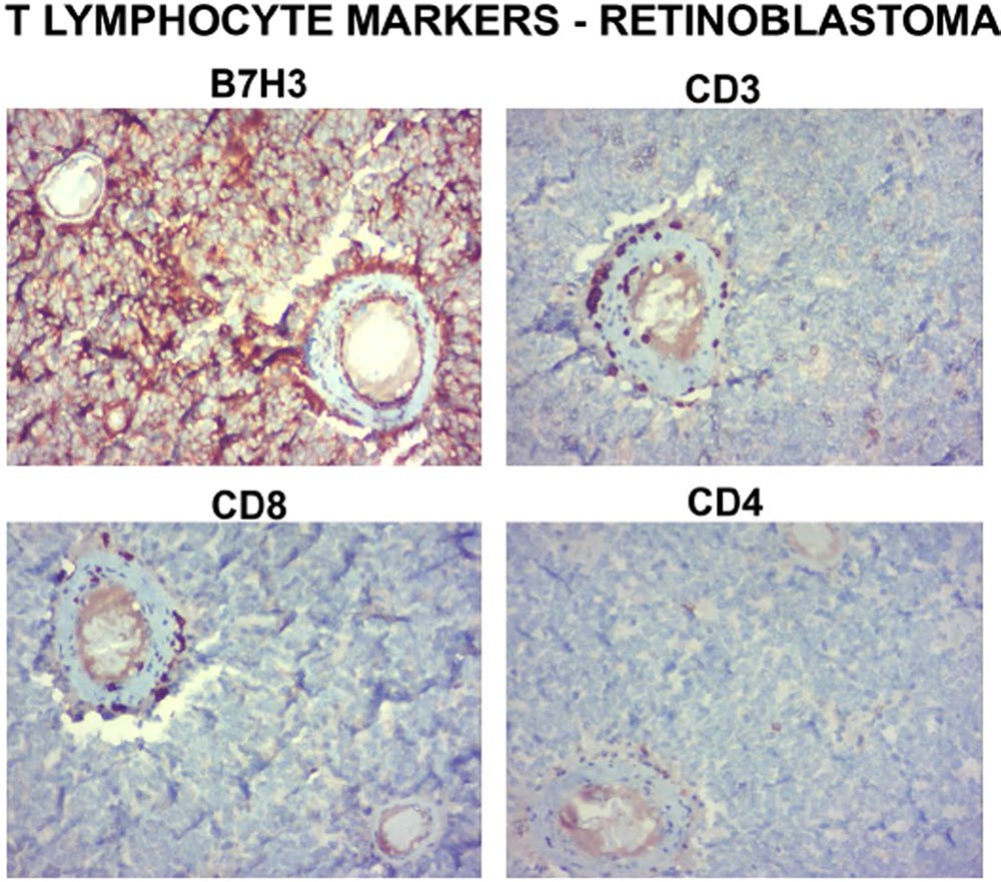
Expression of T lymphocyte markers in RB. B7H3 expression was prominent in the retinoblastoma with sparse distribution of T lymphocytes as revealed by T lymphocyte markers CD3, CD8 and CD4.

CD3, CD4 and CD8 expressing T lymphocytes were found only in the blood vessels near the tumour lobules and retinal regions and very sparsely present in other regions (Fig. 8). In addition to these markers, FOXP3, marker of regulatory T cells were also screened in RB tumors and the expression was almost absent (Supplementary material Fig. S4). In order to understand if the distribution of the T lymphocytes could be related to levels of B7H3, we analysed those blood vessels having higher and lower B7H3 expression. We found that the distribution was related to the B7H3 expression. In addition, since normal cadaveric retina showed B7H3 expression, we wanted to know the distribution of T lymphocyte markers in these tissues. The T lymphocytes were scanty in its distribution even in the normal cadaveric retinal tissue (Supplementary material Fig. S2), suggesting that B7H3 expression and T lymphocyte filtration may be inversely correlated. In order to confirm the finding, we used intraocular tuberculosis section (known to have inflammatory component) and analysed the T lymphocyte distribution in context of B7H3 expression. The results revealed a higher distribution of CD3, CD4 and CD8 T lymphocytes and absence of B7H3 expression (Supplementary material Fig. S5).

## Discussion

Vision salvage still remains an incredible challenge in primary RB treatment. While chemotherapy has been successful in treating RB, pthisical eye due to associated retinal toxicity and chemoresistance of the tumor still exist and lead to enucleation. While the former could be addressed by a targeted approach to chemotherapy, the latter may require newer interventions like immunotherapy. In this study, we analyzed the expression of B7H3 in primary retinoblastoma tissues by immunohistochemistry for two reasons. (i) Our earlier study on proteomics of RB revealed significant expression of B7H3 in primary RB tumor samples compared to normal retina^7^, and, (ii) Definitive role of B7H3 in immune evasion of tumor cells by inhibiting T cell functions.

While the western blotting results confirmed the proteomic study suggesting significantly higher expression of B7H3 in primary RB tumors compared to retina, it was imperative to know the spatial distribution of B7H3 in the context of RB tumor.

The study results suggested that the B7H3 expression was highly heterogenous and it was interesting to see that whenever B7H3 levels were high in the tumor lobules, their levels in the blood vessels confined to the area near these lobules were much reduced and vice-versa. Several studies have reported the expression of B7H3 in both tumor cells and tumor vessels^19^. While differences in the expression patterns of B7H3 between the stromal and tumor cell compartments have been reported, with colorectal and pancreatic cancer showing increased positivity in stroma compared to tumor while, prostate tumors revealing a higher expression in the tumor cells over the stroma; mutually exclusive expression of B7H3 between tumors and blood vessels as observed in RB tumors have not been reported in other cancer types. Whether this observation has any relevance in the nature of the cells in the blood vessels such as endothelial cells or perivascular stromal cells such as pericytes and fibroblasts, or difference in the pre-existing versus newly generated blood vessels require further exploration.

Since, clinical importance of any target molecule in RB tumor rely on certain histopathological features, we studied the expression of B7H3 in the context of the differentiation status of the tumor, site of invasion and degree of anaplasia which were considered relevant for the prognosis. With respect to differentiation status, B7H3 was highly expressed in poorly differentiated RB compared to moderate or well differentiated RB tumors. A retrospective study by Kashyap *et al*. 2012 on 326 primary RB tumors suggested that poorly differentiated tumors was significantly associated with three or more high risk features, specifically massive choroidal invasion compared to well- differentiated tumors^20^. The higher expression of B7H3 in this class of tumors make it highly useful for targeted approaches.

Invasion status of RB tumor determines the region they metastasize. The common regions of metastases are the central nervous system (CNS), regional lymph nodes, bone marrow and the bones^18^. In this study, the sites of invasion were considered as neural and non-neural due to their differences in their prognosis and the regions they metastasize. While neural invasion predominantly lead to CNS metastases; non-neural invasion have higher propensity to metastasize to other systemic sites^21^. It has been reported that CNS metastases have worse prognosis compared to other systemic metastases due to the blood-brain barrier which does not permeate the systemic chemotherapy^19^ and may warrant adjuvant intrathecal or intraventricular chemotherapy^22,23^. B7H3 expression showed a significant increase in the RB tumor invading non-neural tissues compared to neural tissues. It is to be noted that there was no significant difference in the B7H3 expression in the main tumor mass between the two invasions groups, which makes us believe that the B7H3 expression may be suppressed when the tumor invades the optic nerve. At this juncture, this may be perceived as a mere observation, since we could not find any support from published literature. One of the limitations is the number of samples that could be analyzed for such correlation, however, if established with a larger cohort, this finding may have clinical implications using B7H3 as a therapy.

Since, the risk of metastases in retinoblastoma is assessed cumulatively taking into account several factors, we classified the tumors as high-risk and low-risk based on overall histopathological features (as proposed in methodology). B7H3 expression did not vary significantly between these two tumor groups. In addition, recently severe anaplasia has been reported to pose significant risk of metastases in the presence of an otherwise low risk tumor^24^; we also considered comparing low risk tumor with and without severe anaplasia and found no significance between the two groups. The lack of significant difference in the expression is understandable given the different combinations of histopathologic features and heterogenous expression of B7H3 within the tumor.

Since, B7H3 has been reported as both co-stimulatory or co-inhibitory molecule for T lymphocytes, the distribution of T lymphocytes was studied in the RB tumors. Our results showed low numbers CD3, CD4 and CD8 expressing T lymphocytes, restricted to the blood vessels (Supplementary material Fig. S1). The paucity of T lymphocytes in the RB sections prevented us from correlating B7H3 expression with T lymphocyte distribution. However, in order to see if B7H3 expression and presence of T lymphocytes are mutually exclusive, we included intraocular tuberculosis, a well-known inflammatory intraocular condition. The immunohistochemistry revealed an overall absence of B7H3 expression with increased T lymphocytes, which prompts the assumption of a mutually exclusive nature of B7H3 expression and T lymphocyte distribution in intraocular inflammatory conditions (Supplementary material Fig. S2). This might suggest at least in RB, B7H3 may be down-modulating the T cell responses, thereby making it a “cold tumor”. It is also to be noted that the mechanism for lack of T cell infiltration in the cold tumors are not limited to lack of T cell activation alone but may include lack of tumor antigens per se or deficiency of an activated T cell to home into the tumor bed^25^.

Overall, the study reveals an increased, but, heterogenous expression of B7H3 in primary RB tumor samples compared to normal retina. The differences in the spatial distribution of B7H3 expression in the RB tumor prompts us to think the varying strategies required for targeting. While the RB tumors or regions rich in B7H3 could benefit by use of anti-B7H3 drug conjugates^26^ or B7H3 Chimeric Antigen Receptor (CAR)- T cell strategy^27^ lower levels of B7H3 as seen in severe anaplastic regions of RB tumor or the tumor front invading the optic nerve might require an alternative strategy.

The presence of B7H3 in the RB tumour paves way to develop targeted therapies as well as immunotherapy approach as surrogate treatment of the tumour which may be chemo-resistant or to lower chemo toxicity. In addition, it is interesting to see that the B7H3 expression decreases when the tumour enters optic nerve, whether there are any molecules present in the optic nerve tract which decreases B7H3 and their implications in clinical setting is also a probable future study. Although, a long term follow-up was not possible, the clinical data with a follow-up between one and four years did not show any significant correlation with patient survival and B7H3 expression. A longitudinal study with long term follow-up data is essential to understand the relevance of B7H3 expression to prognosticate RB.

## Methods

### Sample collection and ethics approval

The study was complied with declaration of Helsinki and approved by Institutional ethics committee at Vision Research Foundation, (Ethics Number: 563-2016-P) Chennai, India. For western blotting, cadaveric human retinal tissue (control) (n = 4) was obtained from CU shah eye bank of Sankara Nethralaya. Fresh frozen retinoblastoma tumor samples (n = 8) from post therapeutic enucleation submitted for routine histopathological analysis to Larsen & Toubro Ocular Pathology lab were utilized. CU Shah Eye Bank and L and T Ocular Pathology Department are units of Medical Research Foundation. All tissues for research is approved only by the Ethics committee of the organization as per our standard operating procedure.

Retrospective formalin fixed paraffin embedded (FFPE) blocks of RB tumour (n = 35), Intraocular tuberculosis (n = 1) collected between the years 2013–2018 were used in the study for immunohistochemistry. The demographic, clinical and histopathological details are provided in (Table 1). Tumors were divided into three broad categories as high risk (n = 9) and low risk tumors (21), low risk tumors with severe anaplasia (n = 5). (Supplementary Table 1).

**Table 1:** Demographic and histopathological details of primary retinoblastoma tumour samples.

S.No	Age/Sex	Laterality	Differentiation	Invasion	Risk	Anaplasia	IRS score	Follow up(year)*
RB1	2/M	UL	MD	NN	HR	1	12	3
RB2	4/M	UL	MD	N	HR	2.5	9	2
RB3	2/M	UL	PD	N	HR	2	12	3
RB4	4/M	UL	PD	NN	HR	2	9	4
RB5	2/M	UL	PD	NN	LR	2.5	6	3
RB6	3/M	UL	PD	NN	LR	2	9	2
RB7	8/F	UL	PD	NN	HR	0	6	4
RB8	2/M	UL	PD	NIL	LR	2.5	6	3
RB9	3/M	UL	PD	NN	HR	2	4	4
RB10	2/M	UL	MD	N	HR	3	6	3
RB11	0.8/F	UL	MD	NIL	LR	1	6	3
RB12	2/F	UL	WD	NIL	LR	1	2	3
RB13	4/F	UL	PD	NN	LR	3	9	4
RB14	3/F	UL	PD	N	HR	0	12	5
RB15	10/F	UL	WD	NN	HR	0	4	NA
RB16	6/F	UL	MD	NN	HR	3	9	3
RB17	2/F	UL	PD	NN	HR	0	6	2
RB18	3/M	UL	MD	NN	HR	2	9	2
RB19	2/M	UL	WD	NIL	LR	0	4	3
RB20	5/M	UL	WD	NIL	LR	0	4	3
RB21	2/M	UL	MD	NIL	LR	3	9	3
RB22	4/M	UL	PD	N	HR	2.5	9	2
RB23	2/F	UL	MD	NN	HR	1	6	3
RB24	2/M	UL	MD	N	HR	3	9	3
RB25	3/M	UL	PD	NN	LR	2	9	2
RB26	3/F	BL	PD	NN	HR	2.5	9	2
RB27	2/M	UL	PD	NN	LR	2	6	2
RB28	7/M	UL	PD	NN	HR	2	12	NA
RB29	2/M	UL	MD	NN	HR	2	6	3
RB30	3/F	UL	PD	NN	HR	2	12	3
RB31	6/M	UL	PD	NN	LR	0	6	2
RB32	3/M	UL	PD	NN	HR	0	6	1
RB33	2/M	UL	MD	NN	HR	3	4	3
RB34	3/F	UL	PD	NN	HR	2.5	12	1
RB35	4/M	UL	PD	NN	LR	2	9	1

**Abbreviations:** UL-Unilateral, BL-Bilateral, PD-Poorly Differentiated, MD-Moderately Differentiated, WD- Well Differentiated, N-Neural, NN-Non-Neural, LR-Low Risk, HR-High Risk, The numbers indicated in anaplasia 0-No anaplasia, 1- mild anaplasia,2- moderate anaplasia, 2.5- Moderate to severe Anaplasia, 3-Severe Anaplasia. *None of the patients had metastasis, or recurrence and all the patients were surviving till the latest period of follow up.

### Immunoblotting

Proteins were extracted from cadaveric human retina (n = 4; control) and RB tumor (n = 8) using RIPA buffer. The protein concentration was determined by BCA protein assay kit (Thermo Scientific Catalogue No: NY, USA) For separation of proteins, 30- μg protein per well was loaded onto a 10% -SDS-PAGE gel, and run at 110 V. Subsequently, the proteins were transferred to PVDF membranes. The membrane was blocked in 5% dried milk for 1 h at room temperature. Membranes were incubated in primary antibodies against B7H3 (R&D systems AF1027 1 µg/ml), in PBST containing 2% Bovine serum Albumin for overnight at 4 °C. Membranes were washed with PBST and incubated with HRP-conjugated Anti-Goat IgG Secondary Antibody (R& D systems Catalogue No HAF017). The bands in the membrane was detected with clarity western electrochemiluminescence substrate (Biorad; catalogue No: 1705061) under Biorad MP ChemiDoc Imaging systems.

### Immunohistochemistry

Immunohistochemistry was performed to assess the qualitative expression of B7H3 with two different antibodies (BioSB Catalogue No; BSB2810 Rabbit Monoclonal, Santa Barbara CA, USA; R&D systems Catalogue No: AF1027-goat anti Human). Antigen retrieval and the process of IHC was done by Bench Mark GX (Ventana Roche) Automated devices for the Bio-SB antibody. The particular protein was visualized with HRP conjugated anti Rabbit antibody (Sigma Aldrich). The peroxidase was detected with 3,3’ diaminobenzidine (Sigma Aldrich) each section was counter stained with Mayer’s Haematoxylin (sigma Aldrich). Breast Cancer was used as Positive control from S M Surgipath laboratory. All stained sections were analysed under a light microscope (Nikon Eclipse Ci-L, Tokyo Japan). Haematoxylin and Eosin (H and E) Staining of RB Tumor Tissue Sections was performed.

The IHC with the second antibody (R&D systems) done manually and The goat anti Human antibody (5 µg/ml) was incubated for 2 hours and the sections were stained with Anti-Goat HRP-DAB Cell & Tissue Staining Kit (brown; Catalogue No: CTS008) and counterstained with haematoxylin.

Thirty-Five retinoblastoma tumors were used for IHC. In 20 tumor samples (RB1-RB20) the IHC was exclusively carried out by Bio-SB antibody and other 8 tumor samples the IHC was exclusively performed with R& D systems antibody (RB21-RB35) (Table 1). Seven RB tumors were commonly used for IHC with both antibodies. The expression of B7H3 in different area and invasion was analysed to investigate the level of expression. The entire section was initially scanned under 4x objective. All the quadrants of the tumor (apex, towards the lens, choroid Optic nerve) were analysed. Specific controls were included for validation of the immunohistochemistry.

All sections were reviewed by experienced pathologist. The scoring criteria for B7H3 immunostaining were analysed by immunoreactive score (IRS) system. Briefly, category A (intensity of immunostaining) was scored using the following criteria: 0, negative; 1, weak; 2, moderate; 3, strong. Category B (percentage of immunoreactive cells) was scored using the following criteria: 1, (0–25%); 2, (26–50%); 3, (51–75%); and 4, (76–100%). Final scores were calculated by multiplying the scores of categories A and B in the same section; the scores ranged from 0 to 12.

The B7H3 expression was evaluated based on different histopathological features such as the differentiation status, site of invasion, risk features for metastases and anaplasia.

### Statistical analysis

For comparison of two groups either Mann-Whitney U test of student’s t test were applied. Paired or unpaired tests were performed wherever relevant. For comparison of greater than two groups, either One Way ANOVA or Kruskal Wallis was used. The statistical analysis was performed on Graph Pad Prism software and cited wherever relevant.

## Supplementary information

Supplementary information.

## Data Availability

All relevant data are within the paper.
