# Supplementary material for: Clinical relevance of B7H3 expression in retinoblastoma
Source: Sci Rep. 2020 Jun 23;10:10185. doi: 10.1038/s41598-020-67101-7 (PMC7311428; doi:10.1038/s41598-020-67101-7)
Supplement: Supplementary file 1 — Supplementary information. [file 41598_2020_67101_MOESM1_ESM.pdf]

Clinical relevance of B7H3 expression in retinoblastoma

Bhuvaneswari Ganesan[1], Sowmya Parameswaran[2], Ashwani Sharma[3], Subramanian Krishnakumar[1*]

[1] L&T Ocular Pathology Department, Vision Research Foundation, Chennai, India

[2] Radheshyam Kanoi Stem Cell Laboratory, Vision Research Foundation, Chennai, India

[3]Department of Chemistry & Biology, Indian Institute of Science Education and Research(IISER), Tirupati.

***Corresponding Author:**

Dr. Subramanian Krishnakumar, M.D.

Larsen and Toubro Ocular Pathology Department,

Vision Research Foundation,

18,College road, Nungambakkam

Chennai - 600006, India.

Email ID: drkk@snmail.org

drkrishnakumar_2000@yahoo.com

Phone: +91-(044) 28271616 Extn: 1302

Running head: B7H3 expression in retinoblastoma

WESTERN BLOTTING - FULL BLOTS

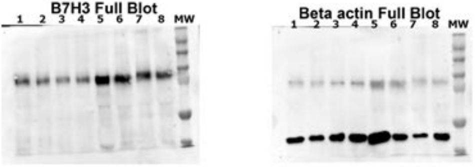

Lanes 1-4 Cadaveric Human retina
5-8 Primary retinoblastoma tumor
MW - Molecular weight marker

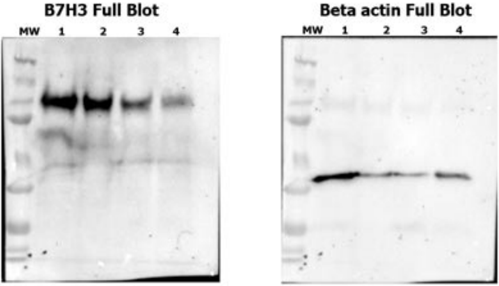

Lanes 1-4 Primary retinoblastoma tumor
MW - Molecular weight marker

Fig. S1. Full blots of B7H3 and Beta-actin on retina and retinoblastoma samples

EXPRESSION OF B7H3 AND T LYMPHOCYTE MARKERS CADAVERIC HUMAN REIINA

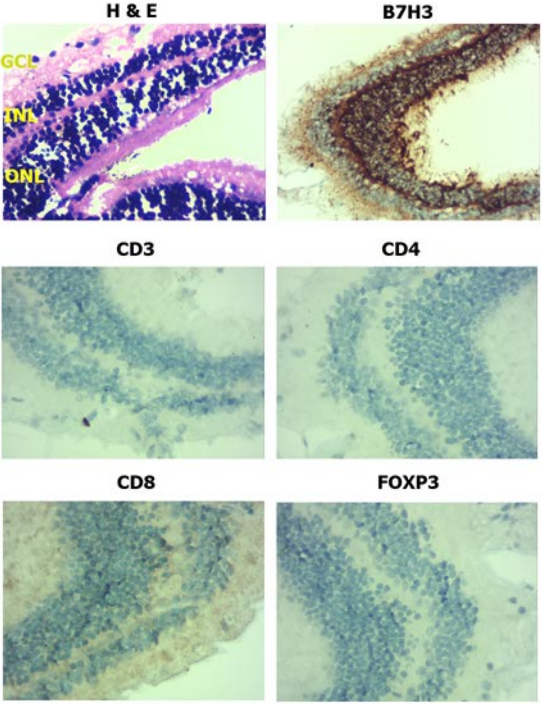

Fig. S2. Expression of B7H3 and T lymphocyte markers in cadaveric human retina. H and E shows the three major celular layers of retina viz. outer nuclear layer (ONL), inner nuclear layer (INL) and ganglion cell layer (GCL). The intensity of B7H3 was highly appreciated in the ONL of cadaveric human retina. T lymphocytes were almost absent as revealed by the absence of CD3, CD4, CD8 and FOXP3 expression in the cadaveric human retina.

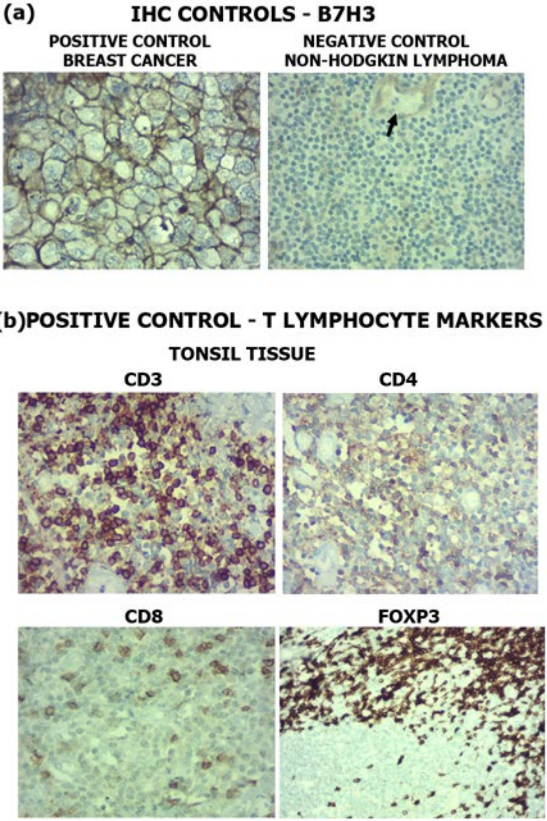

Fig S3. Validation of immunohistochemistry for B7H3 and T lymphocyte markers. (a) B7H3 expression in breast cancer tissue (Positive control) and Non Hodgkin lymphoma (Negative control). Black arrow shows the blood vessels. (b) Expression of T lymphocyte markers CD3, Cd4, CD8 and FoxP3 in tonsil tissue (Positive control).

EXPRESSION OF FOXP3 IN RETINOBLASTOMA

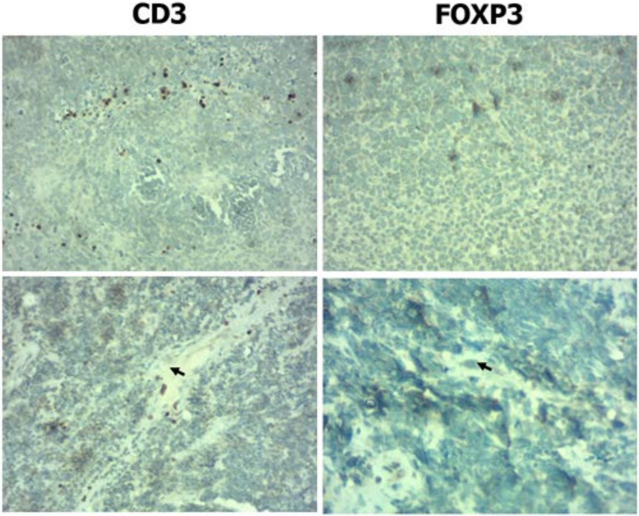

Fig. S4 Expression of FOXP3 in retinoblastoma tumor samples and near blood vessels (black arrow) in the regions positive for CD3 T lymphocytes revealed absence of FOXP3 suggesting lack of T regulatory cells in the tumor.

T LYMPHOCYTE MARKERS - INTRAOCULAR TUBERCULOSIS

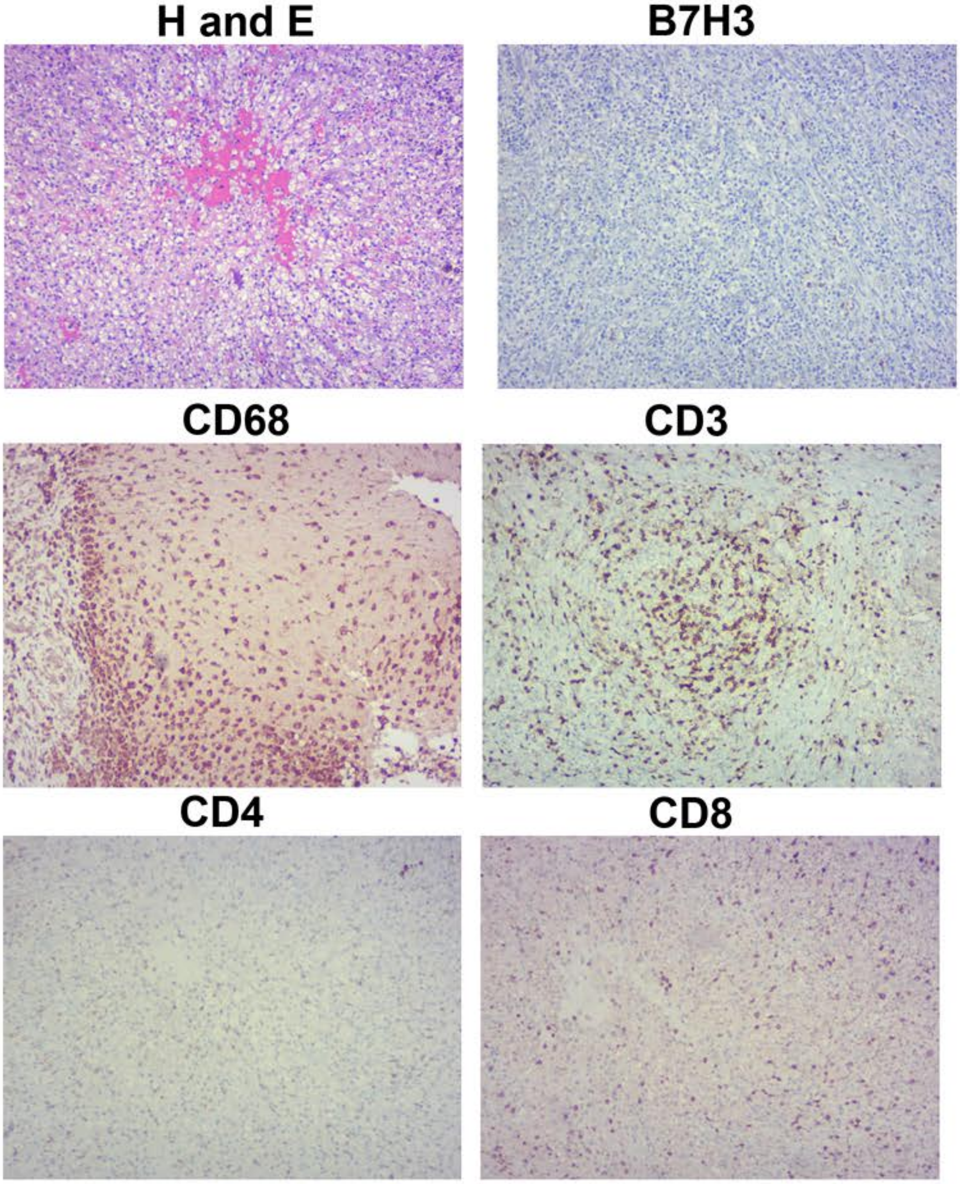

Fig. S5. Expression of B7H3 and T lymphocyte markers in intraocular tuberculosis. Intraocular tuberculosis granuloma revealed lack of B7H3 expression with higher expression of macrophage (CD68) and T lymphocyte (CD3, CD4 and CD8) markers.

Table S1: Criteria of Low and High Risk Tumors

Low Risk Criteria	High Risk Criteria
No Invasion<br><br>Focal Retinal pigment epithelial invasion<br><br>Choroidal invasion <3mm<br><br>Vitreous seeds	Choroidal invasion<3mm with prelaminar/laminar invasion<br><br>Choroidal Invasion>3mm<br><br>Scleral Invasion<br><br>Orbital Invasion<br><br>Post-laminar Invasion of the Optic nerve<br><br>Anterior chamber invasion
